# Supplementary material for: Dynalign II: common secondary structure prediction for RNA homologs with domain insertions
Source: Nucleic Acids Res. 2014 Nov 21;42(22):13939–48. doi: 10.1093/nar/gku1172 (PMC4267632; doi:10.1093/nar/gku1172)
Supplement: SUPPLEMENTARY DATA [file supp_gku1172_nar-02021-z-2014-File012.zip › manual/RNA_class/html/functions_func_0x73.html]

RNAstructure Classes: Class Members - Functions

- Main Page
- Classes
- Files

- Class List
- Class Hierarchy
- Class Members

- All
- Functions
- Variables
- Typedefs

- a
- b
- c
- d
- e
- f
- g
- h
- m
- n
- o
- p
- r
- s
- t
- v
- w
- ~

### - s -

- SetExtrinsic()
  : RNA- SetForbidIntramolecular()
    : HybridRNA- SetIndexSeq()
      : Multilign\_object- SetIterations()
        : Multilign\_object- SetMaxDsv()
          : Multilign\_object- SetMaxPairs()
            : Multilign\_object- SetModifier()
              : RNA- SetNucType()
                : Multilign\_object- SetProgress()
                  : Dynalign\_object
                  , HybridRNA
                  , Multilign\_object
                  , RNA- SetSHAPEIntercept()
                    : Multilign\_object- SetSHAPESlope()
                      : Multilign\_object- SetTemperature()
                        : Multilign\_object
                        , Thermodynamics
                        , TwoRNA- SetupBimolecular()
                          : HybridRNA- single\_z\_predict()
                            : Multifind\_object- SpecifyPair()
                              : RNA- Stochastic()
                                : RNA- StopProgress()
                                  : Dynalign\_object
                                  , Multilign\_object
                                  , RNA
                                  , HybridRNA- storetemplatefilename()
                                    : Dynalign\_object- sum\_multifind()
                                      : Multifind\_object

---

Generated on Tue Jul 30 10:52:29 2013 for RNAstructure Classes by 
 1.5.7.1
